# Alveolar Ridge Split Technique Using Piezosurgery with Specially Designed Tips

**DOI:** 10.1155/2017/4530378

**Published:** 2017-01-29

**Authors:** Alessandro Moro, Giulio Gasparini, Enrico Foresta, Gianmarco Saponaro, Marco Falchi, Lorenzo Cardarelli, Paolo De Angelis, Mario Forcione, Umberto Garagiola, Giuseppe D'Amato, Sandro Pelo

**Affiliations:** ^1^Department of Oral and Maxillofacial Surgery, Catholic University of the Sacred Heart Medical School, Rome, Italy; ^2^Department of Oral and Maxillofacial Surgery, Foligno Hospital, Foligno, Italy; ^3^Department of Oral and Maxillofacial Surgery, Nuovo Ospedale San Giovanni Battista, Foligno, Italy; ^4^Department of Neurosurgery, Queen Elizabeth Hospital, Birmingham, UK; ^5^Department of Orthodontics, University of Milan, Milan, Italy

## Abstract

The treatment of patients with atrophic ridge who need prosthetic rehabilitation is a common problem in oral and maxillofacial surgery. Among the various techniques introduced for the expansion of alveolar ridges with a horizontal bone deficit is the alveolar ridge split technique. The aim of this article is to give a description of some new tips that have been specifically designed for the treatment of atrophic ridges with transversal bone deficit. A two-step piezosurgical split technique is also described, based on specific osteotomies of the vestibular cortex and the use of a mandibular ramus graft as interpositional graft. A total of 15 patients were treated with the proposed new tips by our department. All the expanded areas were successful in providing an adequate width and height to insert implants according to the prosthetic plan and the proposed tips allowed obtaining the most from the alveolar ridge split technique and piezosurgery. These tips have made alveolar ridge split technique simple, safe, and effective for the treatment of horizontal and vertical bone defects. Furthermore the proposed piezosurgical split technique allows obtaining horizontal and vertical bone augmentation.

## 1. Introduction

The treatment of patients with atrophic ridge who need prosthetic rehabilitation is a common problem in oral and maxillofacial surgery. Following the loss of a tooth, the alveolar ridge undergoes bone resorption in the vertical, transversal, and sagittal plane [1]. The majority of the reduction takes place within the first year after the extraction, in particular, within the first three months [1–4]. Initially, there is a greater reduction of the bone thickness rather than in the height. The resorptive process continues throughout the following years; however, the rate of bone loss decreases progressively [5, 6]. The lower jaw is more seriously affected than the upper jaw [6] and the posterior segments of both the mandible and maxilla show more extensive atrophic phenomena compared to the anterior ones [3, 7].

The buccal plate of the upper and lower jaw resorbs more than the palatal/lingual plate. This causes the ridge centre to shift in the lingual/palatal direction [7] but the pattern of bone resorption differs between the upper and lower jaw. While the upper jaw presents an alveolar process wider than the basal bone, in the lower jaw it is the opposite. As a result, the bone resorption of the alveolar process often causes a transversal and sagittal discrepancy.

The bone resorption can make it impossible to insert implants due to inadequate space or can create unfavourable aesthetic and functional conditions for prosthetic rehabilitation. It is well established that implant placement must be prosthetically driven and not bone driven [8].

Bone thickness to allow implant placement should be at least greater than 1.5 mm, both on the vestibular and on the lingual/palatal side [9]. Thus if the alveolar width is less than 6 mm, transversal bone augmentation is generally required to allow implant placement [10–15].

With regard to the vertical plane, a similar success rate has been obtained with implants that have a microrough surface 8 mm long in the lower jaw and 10 mm in the upper jaw [16]. However a substantial level of reliability has also been recorded with the use of short implants. Recently, thanks to the improvements in implant design and surface characteristics, successful results and a low incidence of biological and biomechanical complications have been reported [17].

Among the various techniques introduced for the expansion of alveolar ridges with a horizontal bone deficit is the alveolar ridge split technique. This technique has proved to be a valid procedure and a 98% to 100% survival rate has been reported following the contextual insertion of implants [18–21]. In addition to being an extremely predictable and reliable procedure, the alveolar ridge split technique is characterised by its low invasiveness.

Alternatives to the alveolar ridge split technique are the onlay bone grafts [22], guided bone regeneration [23, 24], and horizontal distraction osteogenesis [25]. The principal disadvantages of onlay bone grafts are the invasiveness, the presence of an additional donor site related to the bone harvesting requirement, and the consistent resorption which the grafted bone undergoes in connection with the chosen donor site [21, 26]. The main problems of the guided bone regeneration are the risk of exposure and collapse of the membranes and the risk of resorption which the grafting material encounters when the membrane is removed [27, 28].

Alveolar ridge split technique was introduced by Tatum Jr. in 1986 with the aim of increasing the amount of bone in the maxilla [29]. This was adapted by Summers in 1994 [30]. Many variations of the ridge split technique have been described by various authors. In 1992 Simion et al. used a longitudinal greenstick fracture in order to extend the socket, performed through osteotomies [31]. In 1994, Scipioni et al. described another variation, whereby a partial thickness flap is created, followed by vertical intraosseous incisions and the simultaneous displacement of the buccal cortical plate, including a portion of cancellous bone, and the implant placement [32].

Alveolar ridge split technique can be carried out by inserting implants simultaneously or it can be done in two steps. A staged split ridge expansion can be used to place implants in atrophic ridges, with the aim of avoiding malfracture of the osteomized buccal plate in the mandible. This technique is accomplished through two surgical procedures, performed six months apart [15, 33]. The first surgical procedure is utilised for bone augmentation and the second procedure for the implant placement. This technique has the same survival rate as one-step split ridge expansion, which is completed in a single surgical procedure [15].

In 2000, Vercellotti et al. introduced piezosurgery in the treatment of the atrophic jaw. Piezosurgery made split technique easier, safer, and also reduced the risk of complications in the treatment of extreme atrophic crests [34–36]. Moreover, the use of piezosurgery has made the success of alveolar ridge split technique less dependent on the surgeon's skills and less affected by the type of procedure chosen [34].

The aims of this prospective study were to (1) determine the reliability of some new tips that have been specifically designed for the treatment of atrophic ridges with transversal bone deficit and (2) evaluate the amount of horizontal and vertical bone gain achieved by means of a two-step piezosurgical split technique, based on specific osteotomies of the vestibular cortex and the use of a mandibular ramus graft as interpositional graft.

## 2. Material and Methods

Fifteen patients were consecutively treated with the proposed new tips and the following technique by our department between January 1, 2012, and May 1, 2015.

Only patients who required vertical and horizontal bone regeneration were selected.

Patients were required to have good oral hygiene prior to treatment. Participants were excluded if they were smokers, they were taking medications known to modify bone metabolism, they were engaged in excessive alcohol consumption, and they had uncontrolled systemic conditions or periodontal disease.

This study was approved by the local ethics committee and all investigations reported have been carried out in accordance with the 1975 Helsinki Declaration, as revised in 2000 for ethical approval. All participants were informed about the objectives and procedures involved in the study and each patient gave informed consent in writing.

Nine patients (six females and three males) were suffering from maxillary atrophy and six patients (two females and four males) had mandibular atrophy.

The procedure was performed under general anaesthesia. At least two hours prior to surgery, 1 g of amoxicillin was administered to all patients. Antibiotic therapy was continued for one week postoperatively. Analgesics were prescribed to manage postoperative pain. Patients were also asked to rinse with chlorhexidine 0.12% twice a day for two weeks following the surgery. All the patients underwent the surgical procedure using the proposed technique.

All the patients underwent radiological examinations prior to surgery. A panorex and a cone beam CT were performed.

Two linear measurements were taken with a calibrated periodontal probe, one during the first surgery (T1) and one during the second surgery (T2): before the alveolar ridge splitting (T1) and at the screws removal (T2). Vertical bone defects were measured from the most apical portion of the bony defect to a line connecting the vestibular cusps or the incisal edge of the teeth adjacent to the site to be augmented. The width of the alveolar ridge was measured at the crestal level. The number of bone blocks, donor sites, and implants placed in each augmented site were recorded. The operative time was also recorded.

Clinical follow-ups were performed at one week, one month, three months, and six months after surgery. A radiological follow-up was performed six months after the operation with a cone beam CT. Further follow-ups ranged between six months and 18 months. Complications during the surgery and in the postoperative time were recorded.

The proposed new tips used were made from stainless steel 420 B. The thickness of the insert base was 1.99 mm and the thickness of the cutting part was 0.70 mm. The tips have two angles, the first one measures 55°, and the second measures 80°.

There are two sets of these tips. The first one is a square shape with sharpened working part edges which are designed to achieve a safe and precise cut in the atrophic alveolar bone. The second set has a blunt profile with sharp edges designed to obtain less sharp lines, to achieve a less aggressive cut as well as to prevent damage to delicate anatomical structures such as Schneiderian membrane or inferior alveolar nerve.

**Figure 1:**
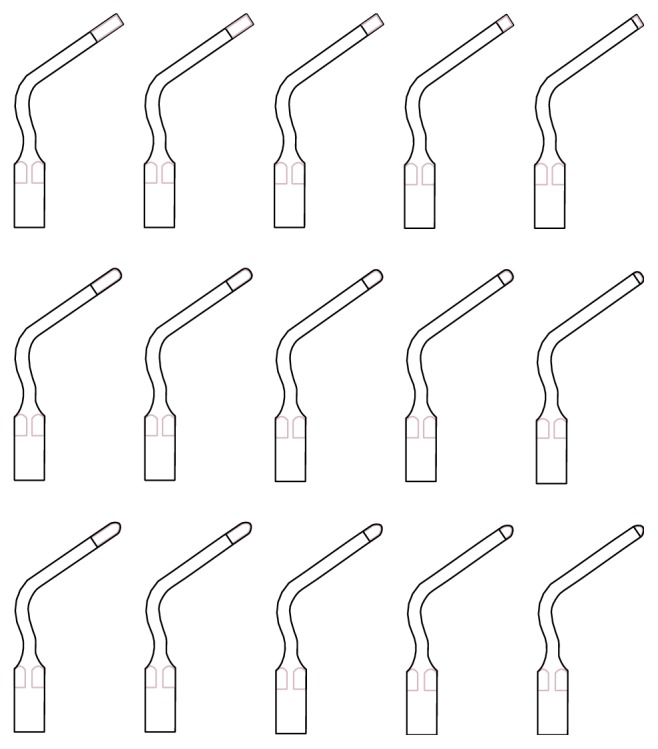
Design of the tips.

Five different inserts of increasing length are available for each set (Figure 1). They are all designed to cut the bone progressively deeper, from 1 mm to 5 mm, each being different from the other by 1 mm. The tips were developed for a piezosurgery unit which offers the ability to set power and regulate vibration function and percussion action (SURGYBONE SILFRADENT®). The variation of these parameters affects the characteristics of the incision.

The tips can be used at high power vibration and also with percussion action, in order to obtain high bone cutting performances and carry out faster osteotomies. However these tips can also be used at low power, without affecting the cutting efficiency, if a precise and delicate bone cut is required to create bone grooves.

## 3. Surgical Technique

Under local or general anaesthesia, a papillary sparing crestal incision is performed on the atrophic ridge. This incision is followed by two vertical releasing incisions beyond the mucogingival line. Then a full thickness mucoperiosteal flap is raised, and when the bone surface is exposed the planned osteotomies are outlined using tip number one at low power, in order to avoid oscillation of the tip and obtain a cut depth 1 mm. Care must be taken to keep the lingual/palatal periosteum attached to the bony surface.

**Figure 2:**
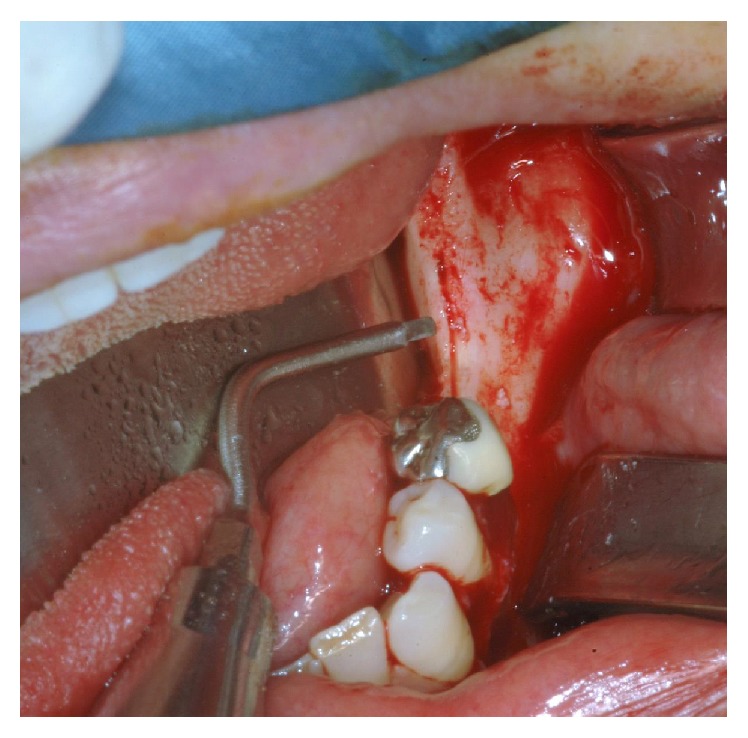
Delicate groove at the centre of the ridge traced setting at low power.

**Figure 3:**
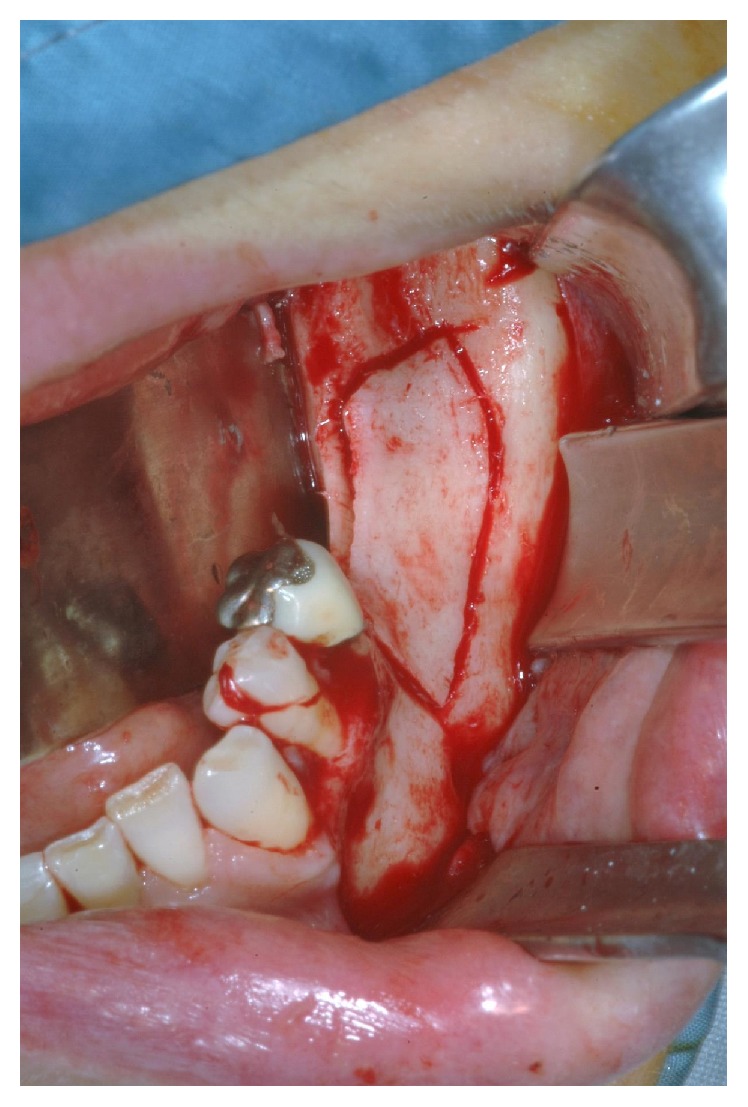
Design of the osteotomies on the mandibular bone.

The first osteotomy is carried out at the centre of the occlusal aspect of the ridge and it is traced, extending the incision in anteroposterior direction for the planned length (Figure 2). Subsequently, the vertical osteotomies are performed on the proximal and distal ends of the crestal incision (Figure 3). In our surgical procedure the vertical osteotomies are convergent and oblique, going from the outer surface of the vestibular cortex to the cancellous bone. In this way the distance between the two vertical osteotomies is greater on the outer side than on the inner side of the vestibular cortical plate. The vertical osteotomies length is determined by the extension of the atrophic ridge.

The osteotomy lines should be traced using the tips progressively in order of size, varying the power level of the characteristics of the incision change too. In this way once the osteotomy lines have been outlined, the tips are used in progression from number one to number five to deepen the osteotomies. As the groove on the bone surface becomes retentive the tips can be used at high power resulting in more aggressive and faster cutting.

The tips are calibrated and this makes it possible to achieve the exact depth of cut desired but, if the cortical width exceeds 5 mm, a normal tip or chisels can be used to complete the osteotomy. In the described surgical procedure, once the desired depth of the crestal and vertical osteotomies are achieved, the caudal ends of the vertical osteotomies are connected by a horizontal incision. This last incision is a partial thickness osteotomy.

The greenstick fracture is made using chisels.

**Figure 4:**
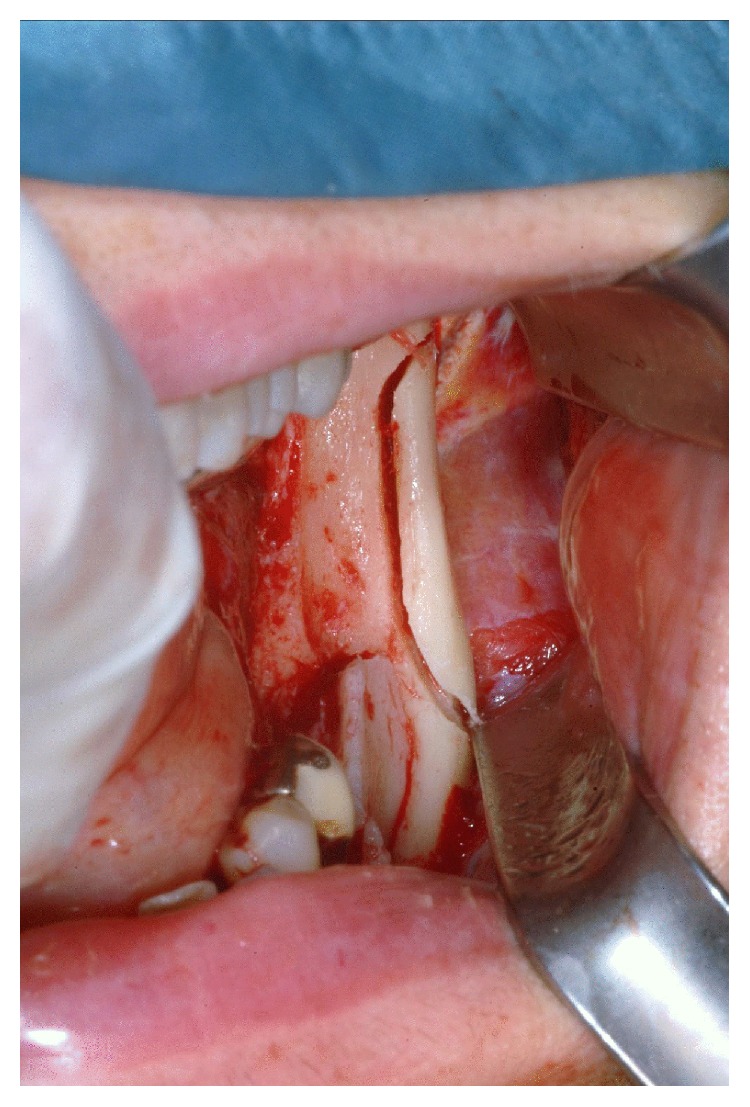
Cortical bone harvesting from the ipsilateral mandibular ramus.

**Figure 5:**
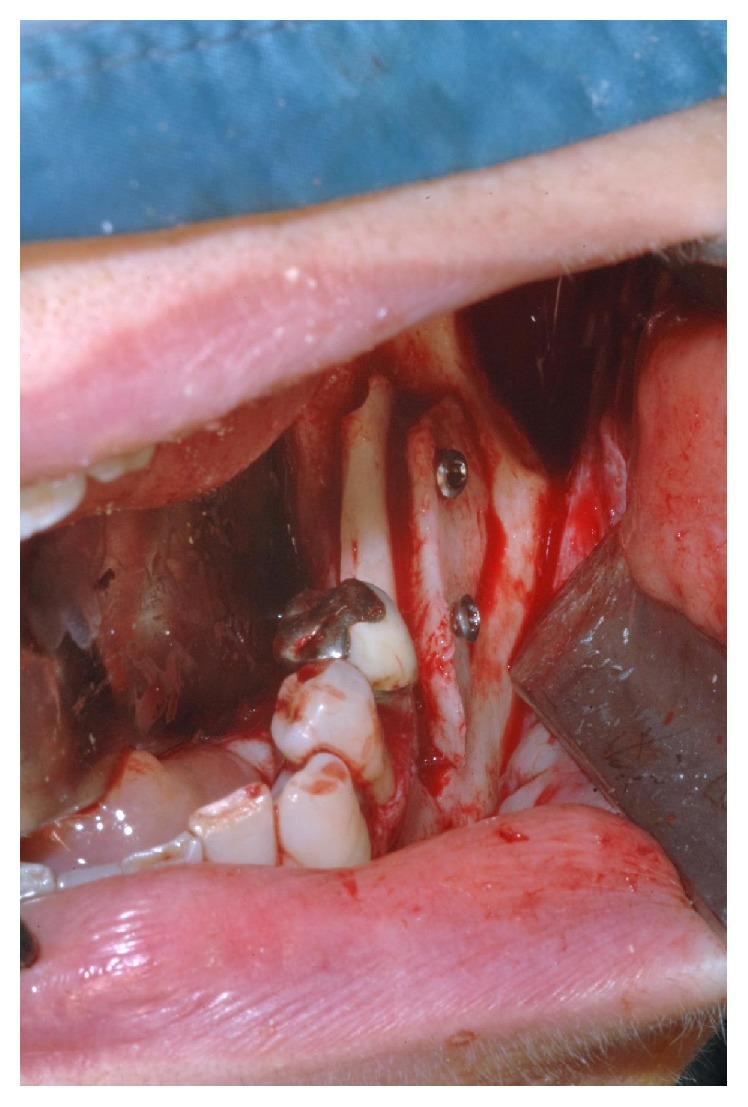
Cortical graft stabilised by means of screws.

A cortical bone graft of appropriate size and shape is harvested from the ipsilateral mandibular ramus by means of the aforementioned tips and chisels (Figure 4). Bone chips can be collected from the same donor site. The cortical graft is gently hammered between the vestibular and lingual cortex, acting as a bone wedge until the desired separation of the two cortices is reached. It is then stabilised using titanium osteosynthesis screws (Figure 5).

**Figure 6:**
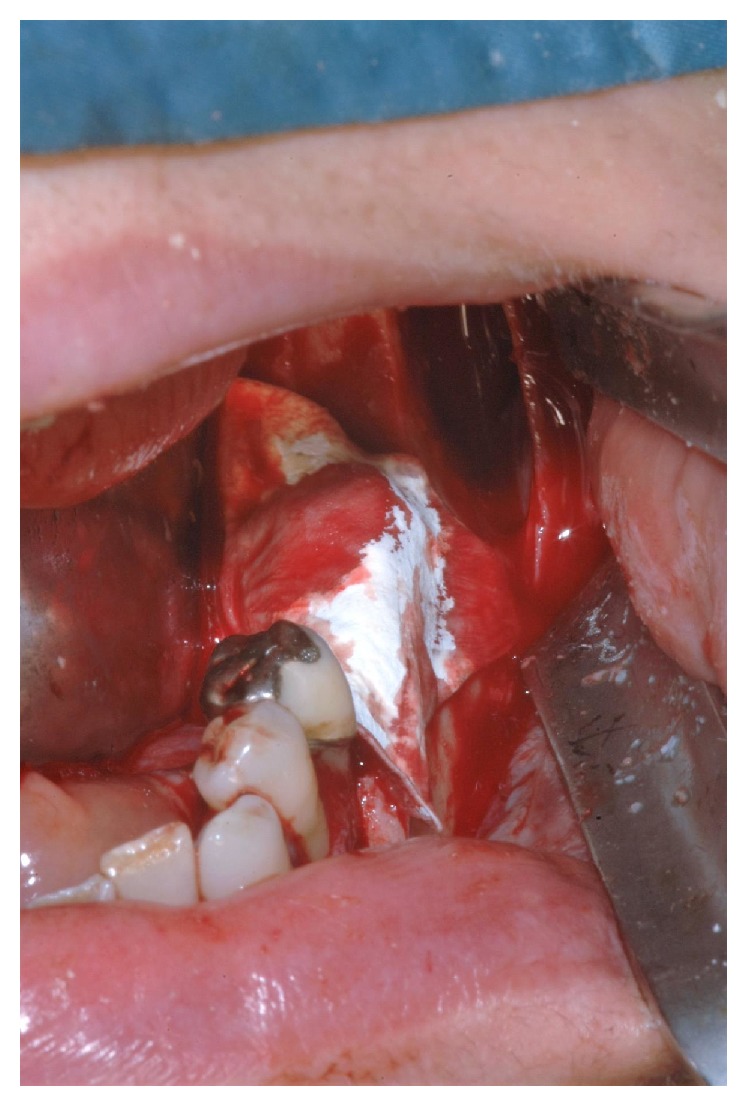
Resorbable membrane covering the grafted site.

In order to obtain supracrestal regeneration the bone graft between the vestibular and lingual/palatal cortices can be fixed at a higher level in order to let it protrude from the occlusal aspect of the two bone plates. In this way the bone graft acts as a vertical support creating a space for the insertion of particulate autografts mixed with bone allograft. Finally the grafted site is covered by a resorbable collagen membrane (Figure 6). The mucoperiosteal flap is repositioned and fixed with 4-0 nonresorbable sutures.

If the buccal segment detaches from the jaw, it can be replaced and stabilised by inserting screws through the graft and the vestibular segment.

The sutures are removed after 10 days. The surgical site is allowed to heal for 6 to 9 months. When healing is complete, the crestal cut is exposed and the screws are removed. Implant beds are conventionally prepared, avoiding damage to the crestal bone, and implants are positioned according to the prosthetic rehabilitation program. Subsequently the submerged implants are exposed and patients receive fixed implant-supported restorations.

## 4. Results

All the patients were partially edentulous. Seven patients were men (47%) and eight were women (53%). Their ages ranged between 35 and 62 years with a mean age of 50.

The average operative time was 54 minutes ranging from 40 to 75 minutes.

Bone regeneration was evaluated at T1 and T2. In general, all treated defect sites exhibited excellent bone formation. The mean vertical augmentation was 3,2 ± 0,4 mm for the mandibular sites and 3,6 ± 0,7 mm for the maxillary sites. The mean lateral augmentation was 5,2 ± 0,7 mm for the mandibular sites and 5,4 ± 1,1 mm for the maxillary sites. Considering all the sites together the mean horizontal and vertical augmentation were, respectively, 5,3 ± 1 mm and 3,4 ± 0,6 mm.

There were no cases of infection and no complications were recorded at the donor sites. A total of two patients developed complications at the recipient sites. There was one early intraoperative complication and one late postoperative complication. The intraoperative complication was a case of vestibular cortex fracture. The postoperative complication was a case of membrane exposure when the alveolar ridge split was associated with a sinus lift, performed using a “monoblock technique.”

All the complications were easily resolved. Soft tissue healing was uneventful and pain and swelling were comparable to usual dentoalveolar procedures.

All the expanded areas were successful in providing an adequate volume to insert implants according to the prosthetic plan. A total of 32 implants were placed.

All implants achieved primary stability and were successful according to the Albrektsson criteria. Prosthetic loading was successfully reached in all cases after the osseointegration of implants.

## 5. Discussion

Alveolar ridge split is a technique for bone expansion used in the treatment of atrophic ridges with horizontal deficits. This technique can be carried out by inserting implants simultaneously or it can be done in two steps. The alveolar ridge split technique with simultaneous implant placement is usually performed to shorten the total treatment time and to eliminate second surgical procedure morbidity [33]. However, there is a higher risk of malfracture of the osteotomized bone segments, especially in the mandible, a lack of initial stability for the implants, and a compromised implant placement in the buccolingual and apicocoronal direction [33]. Among the advantages of the staged alveolar ridge split technique is the ability to insert an interpositional graft, to reduce the risk of uncontrolled fractures in vestibular cortex, and to evaluate the bone augmentation obtained during the second phase of surgery, improved stability, and osseointegration of the implants. Among the disadvantages are the increased morbidity, the duration, and the cost of the therapy.

Alveolar ridge splitting is classically performed by means of chisels and hammer, rotary burs, diamond disk, reciprocal saw [26], or piezoelectric device [33]. The use of bone chisels is time consuming and requires technical skills and a long learning curve [26]. The alveolar ridge split technique performed with burs or rotating saws is more rapid, but soft tissues and delicate anatomical structures can be damaged; close access to adjacent teeth can be difficult, and there is a high risk of losing control over the cutting device. However, the introduction of piezosurgery has enabled the manual instrumentation limits to be pushed, and this makes the procedure a simpler and more reliable technique. The main advantages of the Piezoelectric instrument are a precise and specific cut on mineralised tissues, as well as its capacity to cause minimal tissue damage resulting in improved healing [34, 37]. Furthermore, the introduction of a piezoelectric device for cutting alveolar bone allows this technique to be used regardless of the bone quality [14].

Before carrying out alveolar ridge split technique the patient has to be carefully selected. Good oral hygiene is crucial for the success of the surgery and the prosthetic rehabilitation [38]. Being a smoker should be considered a high failure risk, as, five years after loading, smokers experienced almost twice as many implant failures compared with nonsmokers [39]. Another fundamental and specific requirement for the alveolar ridge split technique is considered the presence of cancellous bone between the two cortices which ensures a good blood supply [40]. This technique is easier to carry out on the upper jaw due to its higher content of cancellous bone and its greater elasticity compared to the mandible [41]. For these reasons, the use of the alveolar ridge split technique requires a minimum bone thickness of 3 mm to 4 mm [42]. Other anatomical requirements are a minimal vertical bone height and no concavity in alveolar bone profile. Finally the horizontal osteotomies have to end at least 1 mm before the neighboring teeth [14].

In order to obtain the most from alveolar ridge split technique and piezosurgery, we have designed new tips to be used with a two steps split technique. These tips have also proved to be extremely useful in other oral and maxillofacial surgical procedures such as bone harvesting, sinus lift, dentoalveolar surgery, and orthognathic and craniofacial surgery.

As a result of our tips and the described procedure, it has been possible to treat atrophic ridges that have less than 3 mm thickness and a small amount of cancellous bone, both in the upper jaw and in the mandible.

These tips were created for delicate osteotomies and in this case are used both in split ridges and for harvesting bone grafts from the mandible.

The tips in the first set are square shaped. This shape was designed to obtain a safe, controlled, and precise cut. The main advantage of this set of tips is the ability to perform faster osteotomies with a high cutting efficiency.

The second set of tips with blunt edges makes it possible to obtain a very precise cut but was specifically designed to be less aggressive and more delicate so that it could be used in the more accurate phases of the osteotomy, avoiding the risk of damaging delicate anatomical structures such as Schneiderian membrane or inferior alveolar nerve.

There are five tips for each of the sets. These are capable of cutting the bone to a depth of 1 mm which can then increase in depth progressively by 1 mm up to a depth of 5 mm.

The 1-2 mm tips are especially useful on low power in order to trace a delicate groove on the bone surface, acting like a marker to draw the osteotomy lines. The shortest tips, with the lowest power, make the most precise cut. Furthermore, the ability to use the shortest tips at a lower power gives the operator increased sensitivity resulting in better control of the cutting device.

The remaining tips used at high power level are faster at cutting, but they remain very precise. These tips should be used once the osteotomy lines have been drawn and a guide is created, to enable a faster and deeper cut.

The tips should be used progressively in order of size, performing repeated shallow grooves to complete the osteotomy and achieve a better result. By using the tips sequentially, it is possible to reach the desired osteotomy depth in a controlled and gradual manner. This allows a measured and extremely accurate progression until the desired depth is reached, without any risk of error or damage to adjacent structures. Furthermore, using the tips in sequence and adhering to this method allow for a bone sparing osteotomy and minimize mechanical stress on the alveolar ridge which avoids undesired fractures being caused in the bone segments.

Access to the anterior and posterior edentulous area is facilitated thanks to the design of the tips.

There are some differences between the proposed piezosurgical split ridge technique and the traditional procedure.

The first difference is the realisation of two vertical osteotomies with an oblique course. The principal determinants of a host-graft union are stability of the construct and contact between host bone and the graft [43]. These vertical osteotomies increase the contact area of the two bone segments, improving the stability and engraftment of the bone graft. Furthermore, there is more space for the insertion of fixation screws in a different location, further away from the planned implant site.

The second difference is in the horizontal osteotomy in connection with the planned axis of rotation for the vestibular cortical plate. With this approach, the location of the greenstick fracture is predetermined. Furthermore, this partial thickness osteotomy prevents any interference with the execution of the greenstick fracture, facilitating the rotation and making it possible to avoid uncontrolled fractures of the vestibular cortical plate.

The described technique is a two-phase procedure. Once the split ridge is complete, a graft of cortical bone is harvested from the ipsilateral mandibular ramus using the proposed tips. The cortical bone graft is positioned between the two cortices and fixed with screws. It has been demonstrated that the application of grafts or bone substitutes in the space between the two cortical bones, together with a membrane, has resulted in a significant reduction in horizontal bone resorption compared to a one-step split technique [44]. The third difference is that, in the proposed technique, if a vertical augmentation is required, the graft between the two cortical bones can be fixed at a higher level in order to let it protrude from the occlusal surface.

Finally the bone graft is stabilised using fixation screws and the primary stability of the bone graft is dependent on adequate screw fixation [45].

Once the bone graft is in position, it creates and maintains space under the membrane around its edges allowing the placement of bone chips mixed with bone allograft which, in turn, promotes further bone regeneration according the principles of the guided bone regeneration.

The guided bone regeneration (GBR) treatment concept advocates that regeneration of osseous defects is predictably attainable via the application of occlusive membranes. These mechanically exclude nonosteogenic cell populations from the surrounding soft tissues, thereby allowing osteogenic cell populations originating from the parent bone to inhabit the osseous wound [46].

The use of barrier membranes in combination with particulate grafts to augment the alveolar ridge and obtain ideal positioning of implants is an effective procedure in both humans and experimental animals [47–50].

In this study, the mean vertical and horizontal augmentation recorded were, respectively, 5,3 and 3,4 mm. The ideal indications of traditional ridge splitting are those sites that do not require vertical ridge augmentation [51]. But, with the described procedure, it is possible to treat transversal bone deficit associated with vertical resorption. The results show an increased bone thickness associated with a considerable vertical augmentation. After the surgery, the incorporation and remodeling of the bone graft create a natural contour of the alveolar ridge and make it possible to use the entire height of the reconstructed bone ridge when inserting the implant.

## 6. Conclusion

The proposed tips help the surgeon to obtain the most from the alveolar ridge split technique and piezosurgery. The main advantages offered are the protection of the delicate anatomical structures, the ability to modulate the depth of the cut, and the precision of the incision, which permits their usage even for the expansion of very thick alveolar ridge. These tips have made alveolar ridge split technique simple, safe, and effective for the treatment of horizontal and vertical bone defects. The proposed tips have also proved to be useful in other surgical procedures such as bone harvesting, sinus lift, dentoalveolar surgery, and orthognathic and craniofacial surgery. Furthermore the use of the described piezosurgical split technique in order to obtain ideal positioning of implants and both horizontal and vertical bone augmentation is an effective procedure.
